# Gastrodin promotes CNS myelinogenesis and alleviates demyelinating injury by activating the PI3K/AKT/mTOR signaling

**DOI:** 10.1038/s41401-025-01492-z

**Published:** 2025-02-26

**Authors:** Xiao-yu Shi, Yi-xi He, Man-yue Ge, Peng Liu, Ping Zheng, Zheng-hao Li

**Affiliations:** 1 State Key Laboratory of Medical Neurobiology and MOE Frontiers Center for Brain Science, Institutes of Brain Science, Fudan University, Shanghai, 200030 China; 2 Department of Neurology, Zhongshan Hospital, Fudan University, Shanghai, 200030 China; 3 Institute of Neuroscience, MOE Key Laboratory of Molecular Neurobiology, Naval Medical University, Shanghai, 200433 China

**Keywords:** demyelinating diseases, myelinogenesis, oligodendrocyte, gastrodin, PI3K/AKT/mTOR, Zebrafish

## Abstract

Demyelination is a common feature of numerous neurological disorders including multiple sclerosis and leukodystrophies. Although myelin can be regenerated spontaneously following injury, this process is often inadequate, potentially resulting in neurodegeneration and exacerbating neurological dysfunction. Several drugs aimed at promoting the differentiation of oligodendrocyte precursor cells (OPCs) have yielded unsatisfactory clinical effects. A recent study has shifted the strategy of pro-OPC differentiation towards enhancing myelinogenesis. In this study we identified the pro-myelinating drug using a zebrafish model. Five traditional Chinese medicine monomers including gastrodin, paeoniflorin, puerarin, salidroside and scutellarin were assessed by bath-application in Tg (MBP:eGFP-CAAX) transgenic line at 1–5 dpf. Among the 5 monomers, only gastrodin exhibited significant pro-myelination activity. We showed that gastrodin (10 µM) enhanced myelin sheath formation and oligodendrocyte (OL) maturation without affecting the number of OLs. Gastrodin markedly increased the phosphorylation levels of PI3K, AKT, and mTOR in primary cultured OLs via direct interaction with PI3K. Co-treatment with the PI3K inhibitor LY294002 (5 µM) mitigated gastrodin-induced OL maturation. Furthermore, injection of gastrodin (100 mg·kg^−1^·d^−1^, i.p.) effectively facilitated remyelination in a lysophosphatidylcholine-induced demyelinating mouse model and alleviated demyelination in the experimental autoimmune encephalomyelitis mice. These results identify gastrodin as a promising therapeutic agent for demyelinating diseases and highlight the potential of the zebrafish model for screening pro-myelinogenic pharmacotherapy.

## Introduction

In the central nervous system (CNS), myelination by oligodendrocytes (OLs) enables saltatory nerve impulse conduction and provides trophic and metabolic support for neuronal axons [1, 2]. Abnormal myelination or demyelination results in cognitive and behavioral deficits in neurological diseases, including multiple sclerosis (MS), leukodystrophies, and some other neurodegenerative disorders [3, 4]. New OLs can be generated throughout life by oligodendrocyte precursor cells (OPCs). After myelin damage, OPCs are recruited to the demyelinated lesion core and differentiated into mature OLs to generate new myelin sheaths, a repair process referred to as remyelination [5].

Although remyelination occurs spontaneously following CNS demyelination, this repair process is often insufficient, which is primarily due to the blockade of OPC differentiation and the reduction in myelinogenic capacity of both surviving and newly differentiated OLs at the demyelinating lesions [6–8]. Even when OPCs differentiate and regenerate myelin sheaths, the newly formed myelin sheaths are shorter and thinner than normal [5]. Therefore, the development of drug screening research to promote OPC differentiation has been encouraged [9]. While a few drugs have finally reached phase 2 clinical trials, only clemastine has shown benefits in the treatment of optic neuritis of MS patients [10]. In response, a recent study has shifted the strategy of pro-OPC differentiation towards enhancing myelinogenesis [6]. A small-molecule epigenetic-silencing-inhibitor was identified to enhance myelin production and ensheathment, highlighting the potential promise of pharmacological intervention for promoting remyelination.

Zebrafish have become a prominent model for investigating myelination in vivo, primarily due to their transparent larval development. Multiple transgenic zebrafish lines and live imaging-based techniques have provided insight into the dynamic biological processes of OL development [11, 12]. As the molecular and cellular functions of zebrafish closely resemble those of mammals, they are considered an optimal tool for efficient screening of phenotype-based drugs [13, 14]. This in vivo model facilitates the observation of biological processes, especially myelin formation. Particularly, transgenic lines that label myelin sheaths, such as Tg(MBP:eGFP-CAAX) [15], provide a valuable tool for screening pro-myelinogenesis drugs.

Traditional Chinese medicine (TCM) monomers are effective single-compound components extracted from Chinese herbal medicine [16]. TCM monomer therapies have been widely used in various CNS diseases, including MS, vascular dementia, and Alzheimer’s disease [17–20]. Previous studies have suggested that TCM monomers have a protective effect against white matter damage [21–23], but whether they could promote myelination remains incompletely understood. Here, we aim to utilize the zebrafish model for screening TCM monomers that are conducive to the treatment of demyelinating diseases.

## Materials and methods

### Animals

All animal experiments were approved by the Scientific Investigation Board of Naval Medical University, the Fudan University Animal Care and Use Committee and were performed in accordance with the National Institutes of Health’s Guide for the Care and Use of Laboratory Animals. C57/BL6 mice and Sprague-Dawley (SD) rats were purchased from Shanghai Jihui Laboratory Animal Co., Ltd. (Shanghai, China), maintained under specific pathogen-free conditions. We anesthetized mice with pentobarbital, and then the whole brains or spinal cords were collected following transcardial perfusion and overnight post-fixation with 4% paraformaldehyde.

Adult zebrafish were husbandry in a recirculating system with a 14:10 light-dark cycle at 28 ± 1 °C [24, 25]. They were provided with plumpy shrimp two times per day. Freshly fertilized eggs were produced by pairwise mating and raised in dish containing system water. The zebrafish lines used in this study included: wild-type AB line, Tg(MBP:eGFP-CAAX) - Membrane eGFP in MBP^+^ cells, Tg(MBP:Gal4) & Tg(5×UAS:GFP) - Gal4 specifically expressed in MBP^+^ cells, and Tg(Olig2:eGFP) - eGFP in Olig2^+^ cells. Tg(MBP:eGFP-CAAX) and Tg(MBP:Gal4) were created by injecting plasmid Sox10:mRFP, MBP:Gal4, MBP:eGFP-CAAX, together with I-SceI enzyme, into one-cell embryos. Tg(5×UAS:GFP) was a gift from Dr Jiu-lin Du (Center for Excellence in Brain Science and Intelligence Technology, Chinese Academy of Sciences, Shanghai, China). Tg(Olig2:eGFP) transgenic line was a gift from Dr B Appel (University of Colorado School of Medicine, Aurora, CO, USA).

### Drug screening

The Tg(MBP:eGFP-CAAX) transgenic line were used for the drug screening. The embryos were mechanically dechorionated at 24 h post fertilization and the larvae were randomly divided into control and the drug-treated groups. Subsequently, they were transferred into small petri dishes containing with 10 μM gastrodin (HY-N0115, MedChemExpress, Monmouth Junction, NJ, USA), paeoniflorin (P0038, Sigma-Aldrich, St. Louis, MO, USA), puerarin (P5555, Sigma-Aldrich, St. Louis, MO, USA), salidroside (43866, Sigma-Aldrich, St. Louis, MO, USA) and scutellarin (73577, Sigma-Aldrich, St. Louis, MO, USA), and solutions were renewed every day during the imaging period. Metformin (D150959, Sigma-Aldrich, St. Louis, MO, USA) was used as a pro-myelinating positive control. The fluorescence-positive larvae were manually selected at 3 days post-fertilization (dpf). The larvae were immobilized in 1.4% low melting-point agarose on a glass coverslip and were positioned on the microscope stage with anterior to left and dorsal to top.

### Single oligodendrocyte labeling

Mosaically labeling oligodendrocytes was performed as previously reported [26]. Fertilized eggs were microinjected at the one-cell stage with 1 nL solution containing 45 ng/μL Sox10:mRFP plasmid DNA, 45 ng/μL Sox10:eGFP plasmid DNA, and I-SceI. Zebrafish were screened at 3 dpf for isolated oligodendrocytes. Sox10:mRFP plasmid was a gift from Dr B Appel (University of Colorado School of Medicine).

### Lysophosphatidylcholine (LPC)-induced demyelinating model

Demyelinated lesions were produced in the corpus callosum white matter of adult mice (8 weeks) as described previously [27]. Mice were deeply anaesthetized with 1.5% isoflurane in 30% oxygen. After exposing the skull, a 34-gauge Hamilton syringe was inserted into the following coordinates: 1.0 mm anterior and 1.0 mm lateral to bregma; 1.9 mm depth from the surface of the skull. 1 μL of 1% LPC (L4129, Sigma-Aldrich, St. Louis, MO, USA) was injected at 0.25 μL/min and the needle was held in position for 5 min to reduce backflow. The mean surface of two adjacent slices (μm^2^) was multiplied by 84 μm (4-μm-thick slice + 80-μm-interslice gap) to calculate the inter-slice volume (μm^3^). The total demyelinated lesion volume (mm^3^) was calculated based on the equation: V = Σ demyelinated lesion area × thickness of the section.

### Experimental autoimmune encephalomyelitis (EAE) induction

EAE induction was performed as previously reported [28]. Briefly, female C57BL/6 mice (8–10 weeks) were subcutaneously immunized with myelin oligodendrocyte glycoprotein peptide 35–55 (MOG 35–55, GL Biochem, Shanghai, China) in complete Freund’s adjuvant containing heat-killed *Mycobacterium tuberculosis* (H37Ra strain; Difco). Pertussis toxin (200 ng/mouse; 516561, Sigma-Aldrich, St. Louis, MO, USA) was administered intraperitoneally day 0 and 2 post-immunization. Clinical EAE scores were examined daily in a blind manner on a scale of 0–5 as follows: 0, no clinical signs; 1, paralyzed tail; 2, paresis; 3, paraplegia; 4, paraplegia with forelimb weakness or paralysis; and 5, moribund state or death.

### Drug administration

For the treatment of LPC mice and EAE mice, 200 μL of gastrodin (100 mg/kg) or vehicle (0.9% NaCl) was injected intraperitoneally daily. The dosage of gastrodin for mice was based on the previous studies [29, 30].

### Cell culture

The detailed methods of the primary OPC culture were previously described [31]. In brief, OPCs were isolated from the cerebral cortex of newborn SD rats, and subsequently cultured in Dulbecco’s modified Eagle’s medium (DMEM, Invitrogen, Waltham, MA, USA) containing 10% fetal bovine serum (FBS, Gibco, Waltham, MA, USA). After 8–10 days growth, the cultures were shaken for 1 h (180 rpm, 37 °C) to remove the microglia, then were shaken for 14 h (200 rpm, 37 °C) to collect the OPCs. OPCs were cultured in cultured DMEM/F12 medium supplemented with PDGFaa (0.1%), B27 (2%), N2 (1%) and BFGF (0.1%) for proliferation, and cultured in neurobasal medium supplemented with 2% B27 for differentiation. Triiodothyronine 10 ng/mL (T3, Sigma-Aldrich, St. Louis, MO, USA) was used as a positive control.

The MO3.13 cell line was purchased from Tongwei (Shanghai Tongwei Industry Co., Ltd, Shanghai, China) and cultured as previously reported [32]. MO3.13 cells were maintained in DMEM supplemented with 10% FBS. When the cells reached a density of 80%–90%, they were trypsinized and seeded into 6-well plates. Differentiation was initiated at 60%–70% confluency, with gastrodin treatment simultaneously.

### Western blot analysis

Total proteins from primary cultured OPCs were extracted using RIPA lysis buffer containing phosphatase and protease inhibitor cocktail on ice. The lysate was centrifuged at 12,000 rpm for 10 min at 4 °C. The supernatant was diluted with 5× loading buffer and boiled at 95 °C for 5 min. Subsequently, the total proteins were subjected to Western blot analysis and incubated with primary antibodies anti-PI3K (1:1000; sc-376112, Santa Cruz Biotechnology, Dallas, TX, USA), anti-p-PI3K (1:1000; 4228, Cell Signaling Technology, Danvers, MA, USA), anti-AKT (1:1000; 9272, Cell Signal Technology, Danvers, MA, USA), anti-p-AKT (1:1000; 4051 and 4060, Cell Signal Technology, Danvers, MA, USA), anti-mTOR (1:1000; 4517, Cell Signal Technology, Danvers, MA, USA), anti-p-mTOR (1:1000; 5536, Cell Signal Technology, Danvers, MA, USA), anti-AMPK (1:1000; ab80039, Santa Cruz Biotechnology, Dallas, TX, USA), anti-p-AMPK (1:1000; 2535, Cell Signal Technology, Danvers, MA, USA), anti-β-catenin (1:1000; 32572, Abcam, Cambridge, UK), anti-MBP (1:500; MAB382, Sigma-Aldrich, St. Louis, MO, USA) at 4 °C overnight. The proteins were visualized and quantified by Image Lab analysis (ODYSSEY CLX, LI-COR Biosciences, Lincoln, NE, USA).

### Immunofluorescence staining

Tissue sections or cells were fixed with 4% paraformaldehyde (PFA), blocked with 10% normal goat serum, and permeabilized 0.5% Triton X-100 in phosphate-buffered saline (PBS). Primary antibodies anti-MBP (1:50; ab7349, Abcam, Cambridge, UK), anti-Sox10 (1:200; AF2864, R&D Systems, Minneapolis, MN, USA), anti-CC1 (1:200; OP80, Sigma-Aldrich, St. Louis, MO, USA), anti-PDGFRα (1:300; AF-307, R&D Systems, Minneapolis, MN, USA), anti-Olig2 (1:500; OB-PGP040-01, Oasis Biofarm, Hangzhou, China), anti-Iba1 (1:200; ab48004, Abcam, Cambridge, UK), anti-GFAP (1:200; ab4674, Abcam, Cambridge, UK) were used at 4 °C overnight, followed by incubation with Alexa secondary fluorescent antibodies (1:200; Jackson ImmunoResearch, Bar Harbor, ME, USA) for 2 h at room temperature. Fluorescence images were captured using fluorescence confocal microscopy (Dragonfly 200, ANDOR, Belfast, Northern Ireland) and quantified using Image-Pro Plus (Media Cybernetics, Rockville, MD, USA).

### RNA isolation, reverse transcription, and quantitative real-time PCR (qPCR)

Total RNA was extracted using Trizol (Invitrogen, Waltham, MA, USA). Each RNA sample was reverse-transcribed into complementary DNA (cDNA) using the HiScript III All-in-one RT SuperMix (Vazyme Biotech Co., Ltd, Nanjing, China). RT-qPCR was performed on a LightCycler 96 apparatus (Roche, Basel, Switzerland) using the SYBR Green Pro Taq HS Premix (Accurate Biotechnology Co., Ltd, Changsha, China). Gene expression was expressed as quantified the mRNA level normalized to that of a standard housekeeping gene (*β-actin*) using the ΔΔCT method and each reaction was set up with three replicates. Primer pairs were as follows: for *β-actin*, F: 5’-GTCCACCGCAAATGCTTCTA-3’; R: 5’-TGCTGTCACCTTCACCGTTC-3’; for *Mbp*, F: 5’-GAGAGCCTGGATGTGATGG-3’; R: 5’-CTGTGCCTTGGGAGGAAG-3’; for *Plp1*, F: 5’-GCACCAAGTTCTGATCCC-3’; R: 5’-TGGCAAAGGCAAAGAGTT-3’.

### 5-Bromo-2’-deoxyuridine (BrdU) incorporation

As described previously [33], for the in vitro experiment, 10 μM BrdU (B5002, Sigma-Aldrich, St. Louis, MO, USA) was added to OPC proliferation medium and incubated for 6 h; for the in vivo experiment, mice were administered BrdU (100 mg/kg) intraperitoneally on a daily basis for a period of 7 days, commencing from 8 days post-lesion (dpl) to 14 dpl. The samples were fixed in 4% PFA and permeabilized with 0.3% Triton X-100, then immersed in 2 M HCl at 37 °C for 30 min and neutralized in 0.1 M sodium borate buffer (pH 8.5) for 20 min. Finally, the samples were incubated with primary anti-BrdU (1:100; ab6326, Abcam, Cambridge, UK) overnight at 4 °C.

### Terminal deoxynucleotidyl transferase dUTP nick end labeling (TUNEL) staining

TUNEL staining was performed using the TUNEL kit (C1089, Beyotime Biotechnology, Shanghai, China) according to the manufacturer’s instruction. OPCs were seeded on the glass slides in differentiation medium for 72 h and fixed before conducting TUNEL staining.

### Luxol Fast blue (LFB) and histological staining

LFB staining was performed to access white matter damage and demyelination [33]. Brain and spinal cord sections were incubated in LFB solution (G1030, Servicebio, Wuhan, China) at 65 °C for 4 h. Excess stain was then removed with distilled water, and the sections were differentiated by placing them in lithium carbonate and cycling in 75% ethanol solution until white matter was distinguished. Finally, the sections were dehydrated with anhydrous ethanol and made transparent with xylene, and sealed with neutral resin. LFB staining images were acquired in the bright field and the area of demyelinating lesions was calculated using ImageJ. For hematoxylin and eosin (H&E) staining, sections were immersed in hydrochloric acid-alcohol for several seconds followed by 1% ammonia water for several seconds, and then placed into Iraqi red dye for 1–3 min. Finally, the slices were dehydrated with ethanol for 5 min and made transparent with xylene for 5 min.

### Molecular docking

Molecular docking was conducted to explore the interaction of gastrodin and phosphatidylinositol 3-kinase (PI3K). The predicted structures of PI3K were generated by Alphafold (DeepMind Technologies, London, UK). The structure of gastrodin was obtained from the PubChem database. The protein was subjected to a series of optimization procedures, including dehydration, hydrogenation, the removal of solvent molecules. AutoDock Tools (The Scripps Research Institute, La Jolla, CA, USA) were applied to prepare and parametrize gastrodin and PI3K. The docking grid documents were generated by AutoGrid of sitemap, and AutoDock Vina (1.2.0) was used for docking simulation [34, 35]. PyMOL (Schrödinger, LLC, New York, NY, USA) software was used to illustrate the PI3K-gastrodin interaction.

### Molecular dynamic simulation

The stability of the chosen docking model according to molecular docking was further conformed by molecular dynamic simulation using the non-commercial version 2022.1 of the Desmond/Maestro molecular dynamics software (Schrödinger, LLC, New York, NY, USA) [36, 37]. TIP3P water molecules were added to the systems, which were then neutralized by 0.15 M NaCl solution. After minimization and relaxation of the system, the production simulation was performed for 100 ns in an isothermal-isobaric ensemble at 300 K and 1 bar. Trajectory coordinates were recorded every 100 ps. The molecular dynamics analysis was performed using Simulation Interaction Diagram from Desmond.

### Cellular thermal shift assay (CETSA)

The CETSA was performed according to the previous studies with a minor modification [38]. In brief, OLs were incubated with 10 µM gastrodin for 3 h following a three-day differentiation period. Subsequently, the cells were trypsinized and distributed into small centrifuge tubes, then they were subjected to a temperature gradient ranging from 39 °C to 49 °C for 3 min using a PCR instrument. The cells were lysed with liquid nitrogen by the freeze-thawing method. The lysate was then centrifuged at 20,000 rpm for 15 min at 4 °C. The supernatant was collected for Western blot analysis.

### Drug affinity responsive target stability (DARTS)

For the DARTS assay, we followed the methodology described in a previous report [39]. The cultured OLs were homogenized using RIPA lysis buffer containing protease and phosphatase inhibitors, followed by centrifugation at 4 °C. The supernatant was collected and the protein content was determined using the BCA quantitative kit (P0010, Beyotime Biotechnology, Shanghai, China). The lysis was then incubated with gastrodin for 20 min. The Proteinase K (100 ng/mL; ST533, Beyotime Biotechnology, Shanghai, China) was then added to each sample for 3 min at room temperature, and the reaction was terminated by heating at 98 °C for 3 min. The samples were then collected for Western blot analysis.

### Transmission electron microscopy (TEM)

According to the previous studies [40], the corpus callosum containing the lesions was dissected after transcardial perfusion and fixed in 2.5% glutaraldehyde at 4 °C overnight. Then the sections were immersed in 1% osmium tetroxide for 45 min, after which they were dehydrated with a graded ethanol series and propylene oxide. They were then flat-embedded in a mixture of propylene oxide and epoxy resin (1:1) within a mould. Semithin sections of 2.5 μm were stained with 1% toluidine blue to find the lesion. Ultrathin sections, 60 nm thick, were mounted on copper grids and stained with uranyl acetate and lead citrate for electron microscopy imaging (Hitachi 7100, Hitachi High-Technologies Corporation, Tokyo, Japan).

### Statistical analyses

The data were analyzed using one-way ANOVA with Tukey *post hoc* test or Dunnett’s multiple comparisons for multiple groups, and unpaired Student’s *t* test or Mann–Whitney U test was used for two groups (details in figure legends). The data are presented as mean ± SEM. The value of *P* < 0.05 was considered statistically significant. All statistical analyses were made using Prism 6 (GraphPad Software, Inc., San Diego, CA, USA) and PASW Statistics 18 (IBM SPSS Statistics, Armonk, NY, USA).

## Results

### Drug screening in zebrafish identifies gastrodin as a pro-myelinating agent

**Fig. 1:**
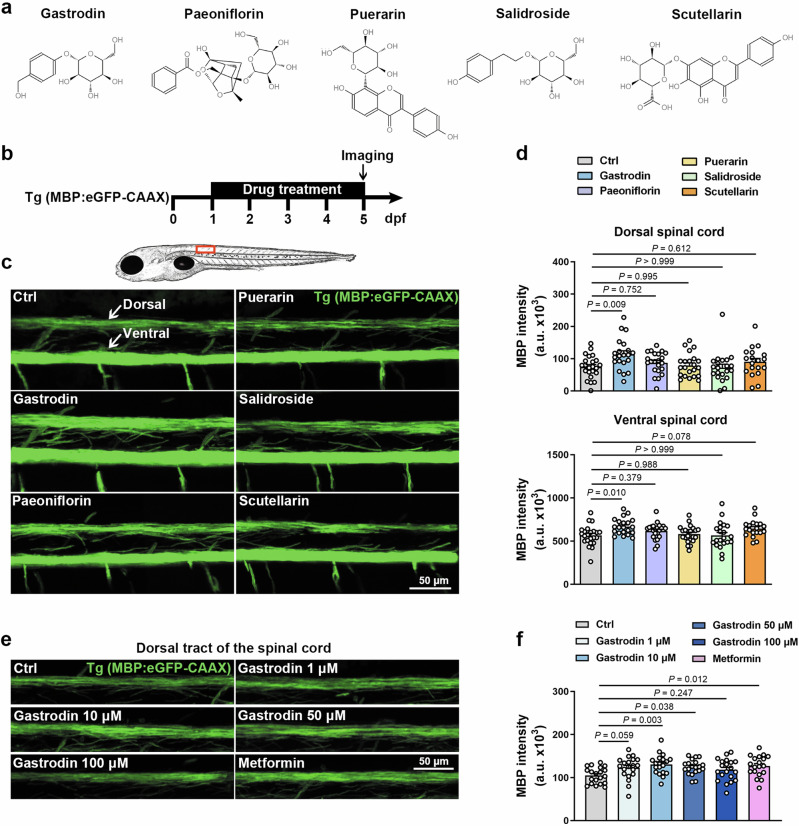
Drug screening in zebrafish identifies gastrodin as a pro-myelinating agent. **a** Chemical structure of the TCM monomers (from ChemicalBook). **b** Schematic of drug screen pipeline. **c** Representative images depicting lateral view of the spinal cord in Tg(MBP:eGFP-CAAX) line at 5 dpf. Red box: location of zoomed-in example region (around 8–11 somites). **d** Mean fluorescence intensity of eGFP fluorescent intensity in the dorsal (above) and ventral (below) tracts of the spinal cord (*n* = 19–23 zebrafish). **e** Representative images depicting the dorsal tracts of the spinal cord in Tg(MBP:eGFP-CAAX) line at 5 dpf. Metformin serves as positive controls. **f** Mean fluorescence intensity of eGFP fluorescent intensity in the dorsal tracts of the spinal cord (*n* = 20 zebrafish). Data are represented as mean ± SEM, One-way ANOVA and Dunnett-*t* in **d** and **f**.

Myelination in zebrafish spinal cord begins at approximately 2.5 days post fertilization (dpf), first ventrally and then dorsally, and myelin sheath is almost stable by 5 dpf [11]. Myelin formation can be visualized via Tg(MBP:eGFP-CAAX) transgenic line, in which membrane-tethered eGFP fluorescence intensity is a proxy measure of *Mbp* promoter activation. To identify the pro-myelinating drug, we evaluated five TCM monomers, including gastrodin, paeoniflorin, puerarin, salidroside and scutellarin (Fig. 1a), by bath-application of Tg(MBP:eGFP-CAAX) line at 1–5 dpf (Fig. 1b). No morphological abnormalities were observed by TCM monomers treatment (Supplementary Fig. S1a). Notably, in comparison with the control group, a significant increase in eGFP fluorescence intensity was observed at 5 dpf in the dorsal and ventral tracts of the spinal cord following gastrodin treatment, but not in the other groups (Fig. 1c, d). Subsequently, the effects of concentration gradients of gastrodin on myelination were tested for optimal dosage, and metformin was selected as a positive control drug. Previous studies and our previous report have demonstrated that metformin has a significant pro-myelinating effect [41, 42]. The eGFP fluorescence intensity in the dorsal tracts of the spinal cord showed that the administration of both 10 µM and 50 µM gastrodin resulted in the promotion of myelination, with the more pronounced effect observed at 10 µM (Fig. 1e, f). Therefore, these data indicate that gastrodin facilitates myelination in the zebrafish spinal cord during the developmental stage.

### Gastrodin promotes myelination by increasing myelin sheath formation

Oligodendroglial development is involved in the process of OPC migration, proliferation, differentiation, and myelin sheaths formation. To further determine the targeting stage of gastrodin, we first examined the number of oligodendrocyte linage cells by Tg(Olig2:eGFP), which labels OL lineage cells ranging from OPCs to myelinating OLs (Supplementary Fig. S1b). There was no difference in the number of dorsal Olig2^+^ cells between gastrodin-treated larvae and control at 3 and 5 dpf (Supplementary Fig. S1c, d), suggesting gastrodin has no effect on the number of OL lineage cells.

**Fig. 2:**
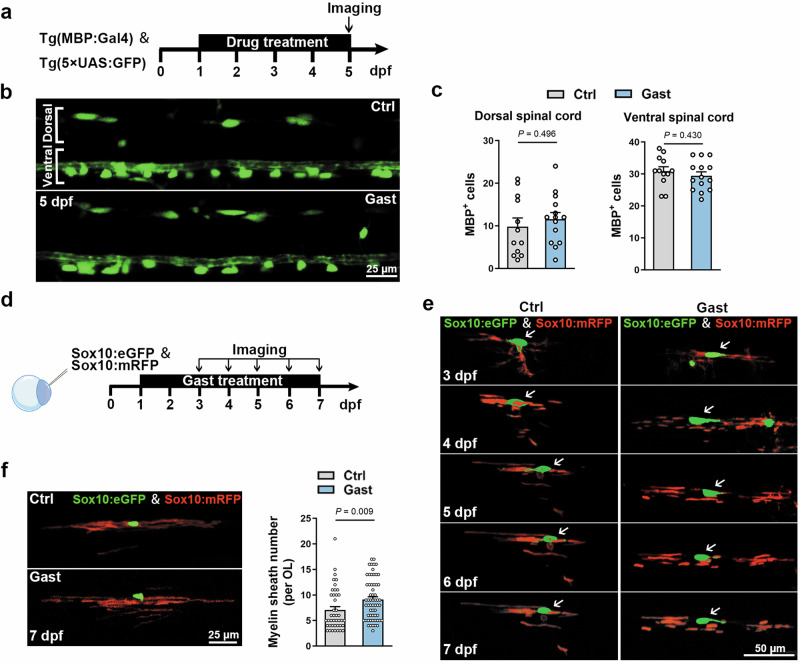
Gastrodin promotes myelin sheath formation. **a** Schematic of Tg(MBP:Gal4) & Tg(5×UAS:GFP) imaging pipeline. **b** Representative images showing lateral views of the spinal cords at 5 dpf. **c** Quantification of GFP^+^ cells number in the ventral or dorsal spinal cord (*n* = 12–14 zebrafish). **d** Schematic of DNA microinjection and imaging pipeline. **e** Time-lapse imaging of myelin sheath development of a single OL from 3 dpf to 7 dpf. **f** Representative images showing myelin sheaths formed by a single OL at 7 dpf and quantification of myelin sheath number per OL (*n* = 42–60 OLs). Data are represented as mean ± SEM, unpaired Student’s *t* test in **c** and **f**.

Furthermore, by crossing Tg(MBP:Gal4) with Tg(5xUAS:GFP), in which OLs were labeled by GFP, we also found that gastrodin did not affect the number of MBP^+^ mature OLs (Fig. 2a–c). To further investigate the myelinating capability of individual oligodendrocytes, we injected both sox10:mRFP and sox10:eGFP plasmids into fertilized eggs at the one-cell stage to mosaically label individual OLs (Fig. 2d). The red fluorescence labeled the membrane and the green fluorescence labeled the cell body (Fig. 2e). Time-lapse imaging showed the dynamic development of a single representative OL from 3 dpf to 7 dpf, and revealed that myelin fragments were more abundant formed by the gastrodin-treated OL (Fig. 2e). Furthermore, the average number of myelin sheaths per OL was increased dramatically in gastrodin-treated larvae at 7 dpf (Fig. 2f). Thus, these collective evidences indicate that gastrodin promotes myelination primarily by enhancing the myelinogenic capacity.

### Gastrodin promotes OL maturation

**Fig. 3:**
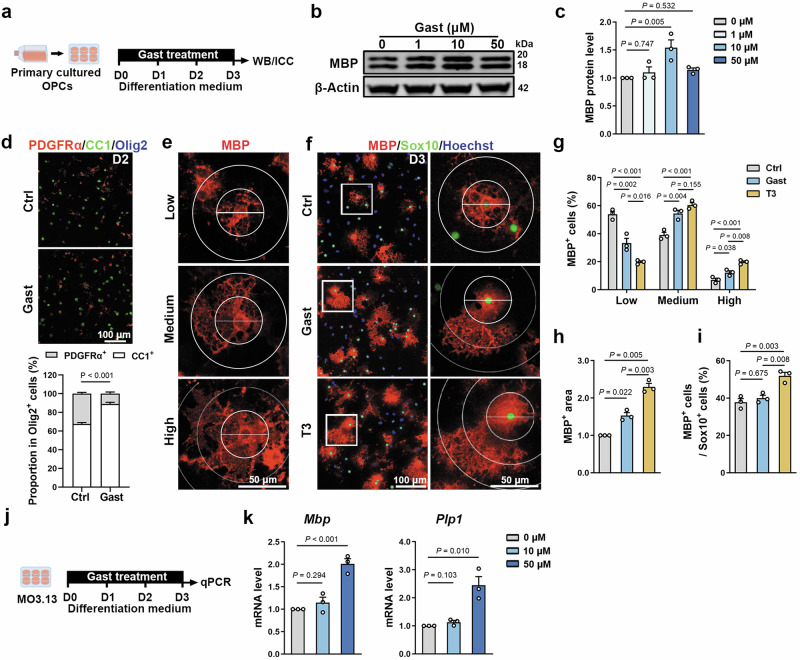
Gastrodin promotes OL maturation in vitro. **a** Schematic depicting OPCs culture and gastrodin treatment. **b** Western blot for MBP after 1, 10, 50 μM gastrodin treatment. **c** Quantification of relative MBP protein levels (*n* = 3 experiments). **d** Representative images of PDGFRα (red), CC1 (green), and Olig2 (blue) immunofluorescence and quantification of proportion of CC1^+^ cells or PDGFRα^+^ cells in Olig2^+^ cells (*n* = 3 experiments). **e** Representative images of OLs membrane area of MBP^+^ cells, depicting different degrees of OL maturation. Low differentiation, OLs without membrane; Medium differentiation, OLs with a few membranes; High differentiation, OLs with large area of membrane. **f** Representative images of MBP (red) and Sox10 (green) immunofluorescence. White boxes zoom in are shown on the right. **g** Quantification of MBP^+^ cells’ proportion in different maturation degrees (*n* = 3 experiments). **h** Quantification of MBP^+^ area (*n* = 3 experiments). **i** Quantification of proportion of MBP^+^ cells in Sox10^+^ cells (*n* = 3 experiments). **j** Schematic depicting MO3.13 oligodendroglial cell line culture and gastrodin treatment. **k** qPCR analysis of *Mbp* and *Plp1* after 10 and 50 μM gastrodin treatment (*n* = 3 experiments). Data are represented as mean ± SEM, One-way ANOVA and Dunnett-*t* in **c**, unpaired Student’s *t* test in **d**, **g**–**i**, and **k**.

Next, we assessed the role of gastrodin in primary cultured OPCs (Fig. 3a). After treatment with different concentrations of gastrodin, we found that 10 μM gastrodin led to a greater increase in MBP expression compared to other concentrations (Fig. 3b, c). On the second day of in vitro differentiation, gastrodin promoted the differentiation of a larger proportion of PDGFRα^+^ OPCs into CC1^+^ OLs (Fig. 3d).

OPC differentiation into mature OLs is accompanied with expansion of cell branching complexity and membrane morphology [43]. As shown in Fig. 3e, MBP staining displayed different degrees of OL maturation. We found that gastrodin significantly enhanced OLs membrane expansion and sheet formation on the third day of in vitro differentiation (Fig. 3f). Triiodothyronine (T3), an effective inducer of OPC differentiation and maturation [44], was used as a positive control. Morphometric and quantitative analysis showed that the branching complexity of OLs was generally higher, and MBP^+^ membrane area increased after gastrodin treatment (Fig. 3g, h). However, there was no difference in the proportion of MBP^+^ cells in OLs after gastrodin treatment (Fig. 3i), indicating that gastrodin primarily impacts the differentiation status of OL, rather than the number of mature OLs. In addition, gastrodin treatment did not affect OPC proliferation or apoptosis as revealed by 5-Bromo-2’-deoxyuridine (BrdU) and terminal deoxynucleotidyl transferase dUTP nick end labeling (TUNEL) staining (Supplementary Fig. S2a–e).

To test whether gastrodin promotes human oligodendrocyte differentiation, we treated MO3.13 human OLs using 10 μM and 50 μM gastrodin (Fig. 3j). qPCR analysis showed that 50 μM gastrodin significantly increased the levels of *Mbp* and *Plp1* (Fig. 3k). Together, these observations confirm that gastrodin promotes OL maturation in vitro.

### Gastrodin activates PI3K/AKT/mTOR signaling pathway in OLs

**Fig. 4:**
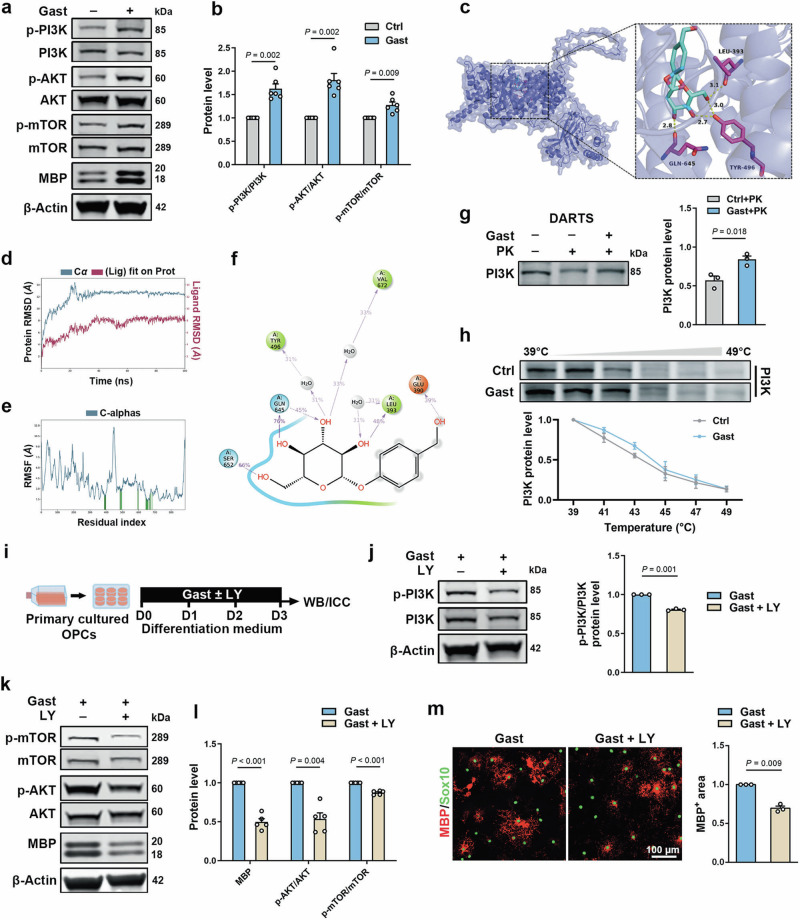
Gastrodin activates PI3K/AKT/mTOR signaling pathway in OLs. **a** Western blot for p-PI3K, PI3K, p-AKT, AKT, p-mTOR, mTOR and MBP. **b** Quantification of the ratio of p-PI3K/PI3K, p-AKT/AKT, and p-mTOR/mTOR (*n* = 6 experiments). **c** The predicted binding model of gastrodin-PI3K complex by molecular docking. **d** Molecular dynamic simulation showing the plot of RMSD (in ångstrom) of the complex during 100 ns MD simulation. **e** RMSF values of PI3K during molecular dynamic simulations. Protein residues that interact with the ligand are marked with green-colored vertical bars. **f** A schematic of detailed ligand atom interactions with the protein residues. Interactions that occur for more than 30.0% of the simulation time in the selected trajectory (0.00 to 100.00 ns) are shown. **g** Western blot and quantification of relative PI3K protein levels (*n* = 3 experiments). Cell lysis was incubated with gastrodin and then subjected to proteinase K (PK). **h** Cellular Thermal Shift Assay, effect of gastrodin on the thermal stability of PI3K and quantification of relative PI3K protein levels (*n* = 3 experiments). **i** Schematic depicting OPCs culture with gastrodin and LY294002 (LY) treatment. **j** Western blot for p-PI3K and PI3K and quantification of the ratio of p-PI3K/PI3K (*n* = 3 experiments). **k** Western blot for p-mTOR, mTOR, p-AKT, AKT, and MBP. **l** Quantification of relative MBP protein levels and the ratio of p-mTOR/mTOR and p-AKT/AKT (*n* = 5 experiments). **m** Left: Representative images of MBP (red) and Sox10 (green) immunofluorescence. Right: Quantification of MBP^+^ area (*n* = 3 experiments). Data are represented as mean ± SEM, unpaired Student’s *t* test in **b**, **g**, **j**, **l**, and **m**.

Previous studies have demonstrated that the phosphatidylinositol 3-kinase (PI3K), adenosine 5’-monophosphate (AMP)-activated protein kinase (AMPK), and Wnt signaling pathways are involved in regulation of myelination [41, 45]. To elucidate the specific signaling pathway underlying the promotion of OL maturation by gastrodin, we examined the expression of key proteins along the above signaling pathways. Western blot showed that gastrodin did not affect the levels of β-catenin and AMPK phosphorylation in the OLs (Supplementary Fig. S3a–c). While phosphorylation levels of PI3K, protein kinase B (PKB, aka AKT), and mammalian target of rapamycin (mTOR) were significantly increased in the gastrodin-treated OLs (Fig. 4a, b), indicating that gastrodin activated the PI3K signaling pathway.

To gain further structural insight into the mechanism by which gastrodin regulates the PI3K signaling pathway, molecular docking was performed to predict whether there is a direct interaction between gastrodin and PI3K. The analysis revealed that gastrodin binds to the PI3K with an affinity of −7.1 kcal/mol, primarily through hydrogen bonds at LEU393, TYR496, GLN645 (Fig. 4c). Dynamic simulation confirmed the stability of this interaction, with the complex stabilizing in 100 ns, as indicated by the root mean square deviation (RMSD) results (Fig. 4d). The RMS fluctuations (RMSFs) during the simulation were plotted to further assess the local structural flexibility. The minimal fluctuations in majority of amino acids suggested strong binding (Fig. 4e). In addition, multiple sets of forces such as hydrogen bonds and water bridges were formed between gastrodin and PI3K (Fig. 4f). Drug affinity responsive target stability (DARTS) revealed that the proteolysis of PI3K protein by proteinase K was attenuated in the presences of gastrodin (Fig. 4g). Correspondingly, cellular thermal shift assay (CETSA) demonstrated that gastrodin also increased the thermal stability of PI3K protein (Fig. 4h). These results support the potential of PI3K as a target of gastrodin in OLs and provide insight into its mechanisms of action.

We then co-treated OPCs with gastrodin and the PI3K inhibitor LY294002 (5 µM). LY294002 could effectively reduce the phosphorylation level of PI3K (Fig. 4i, j). Meanwhile, LY294002 also inhibited the phosphorylation levels of AKT and mTOR (Fig. 4k, l). In particular, the MBP protein level was significantly reduced in gastrodin-treated OLs after LY294002 treatment, as confirmed by immunofluorescence staining results showing that the area of MBP fluorescence was also reduced by LY294002 (Fig. 4k–m). Taken together, gastrodin promotes OL maturation by activating the PI3K/AKT/mTOR signaling pathway, probably through a direct interaction with PI3K.

### Gastrodin promotes remyelination after LPC-induced demyelination

**Fig. 5:**
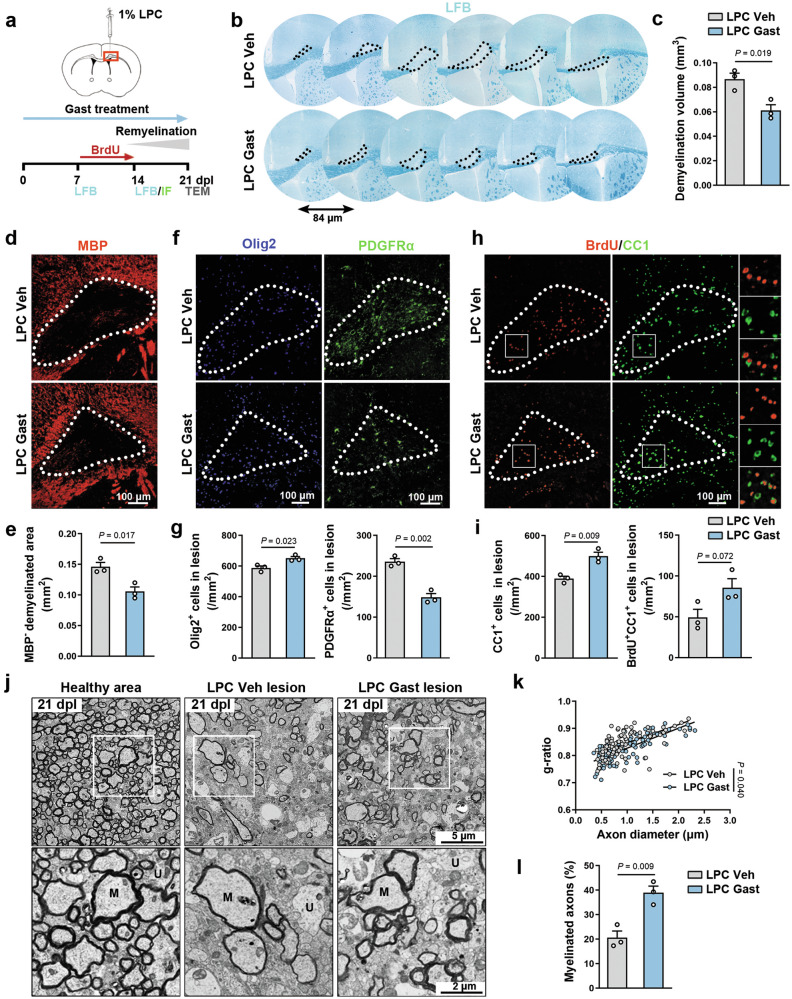
Gastrodin promotes remyelination after LPC-induced demyelination. **a** Schematic depicting LPC-induced demyelination model. **b** LFB staining showing the serial demyelinating region in the corpus callosum. Lesions are shown in the black dotted line. **c** Quantification of the demyelination volume at 14 dpl (*n* = 3 mice). **d** Representative images of MBP (red) in the core lesion. Lesions are shown in the white dotted line. **e** Quantification of MBP^-^ demyelinated area (*n* = 3 mice). **f** Representative images of Olig2 (blue) and PDGFRα (green) in the core lesion at 14 dpl. Lesions are shown in the white dotted line. **g** Quantification of Olig2^+^ cells/mm^2^ and PDGFRα^+^ cells/mm^2^ in the core lesion (*n* = 3 mice). **h** Representative images of BrdU (red) and CC1 (green) immunofluorescence in the core lesion at 14 dpl. Lesions are shown in the white dotted line, and white boxes zoom in are shown on the right. **i** Quantification of CC1^+^ cells/mm^2^ and BrdU^+^CC1^+^ cells/mm^2^ in the core lesion (*n* = 3 mice). **j** TEM images of the LPC-lesioned corpus callosum at 21 dpl. White boxes zoom in are shown below. M Myelinated axon, U Unmyelinated axon. **k**, **l** Quantification of g-ratio and proportion of myelinated axons in the LPC-lesioned corpus callosum at 21 dpl (*n* = 3 mice). Data are presented as mean ± SEM, unpaired Student’s *t* test in **c**, **e**, **g**, **i**, **k**, and **l**.

To examine whether gastrodin promotes remyelination in vivo, we used a lysophosphatidylcholine (LPC)-induced demyelination model [46]. Several animal studies based on gastrodin have shown that 100 mg/kg is a commonly used and effective dosage. Wang et al. found that gastrodin (100 mg/kg) suppresses neuroinflammation and microglial activation in LPS-treated mice [29]. Song et al. also reported that gastrodin (100 mg/kg) might alleviate the LPS-induced neuroinflammation and depressive-anxiety-like behaviors in mice [47]. Therefore, we test the effect of 100 mg/kg gastrodine in the LPC model. LPC injury was induced in the corpus callosum of adult mice that were treated with gastrodin or vehicle daily (Fig. 5a). Apparent demyelinating lesions were shown at 7 days post-lesion (dpl), but there was no significant difference in the demyelinating area between gastrodin-treated mice and vehicle-treated mice (Supplementary Fig. S4a, b).

It has been reported that remyelination starts at around 14 dpl [46]. Compared to the vehicle-treated mice, markedly reduced demyelination volume was observed in the gastrodin-treated mice at 14 dpl (Supplementary Fig. 5b, c). Immunofluorescence staining of MBP revealed the demyelinated area at the core lesion (LPC injection site) was smaller in gastrodin-treated mice at 14 dpl (Fig. 5d). Besides, the gastrodin-treated lesions exhibited a greater number of Olig2^+^ cells and a reduction in PDGFRα^+^ cells at 14 dpl (Fig. 5f, g). We then administered BrdU intraperitoneally to mice from 8 dpl to 14 dpl. The results demonstrated a notable elevation in CC1^+^ cells within the lesions following gastrodin treatment. Additionally, the number of BrdU^+^CC1^+^ cells (newly formed mature OLs) was higher in the gastrodin-treated mice, although this difference was not statistically significant (Fig. 5h, i). Moreover, transmission electron microscopy indicated significantly thicker myelin sheaths and higher proportion of myelinated axons in the gastrodin-treated lesions at 21 dpl (Fig. 5j–l). These observations suggest that gastrodin treatment effectively facilitates the remyelination process in vivo.

### Gastrodin enhances remyelination in EAE mice

**Fig. 6:**
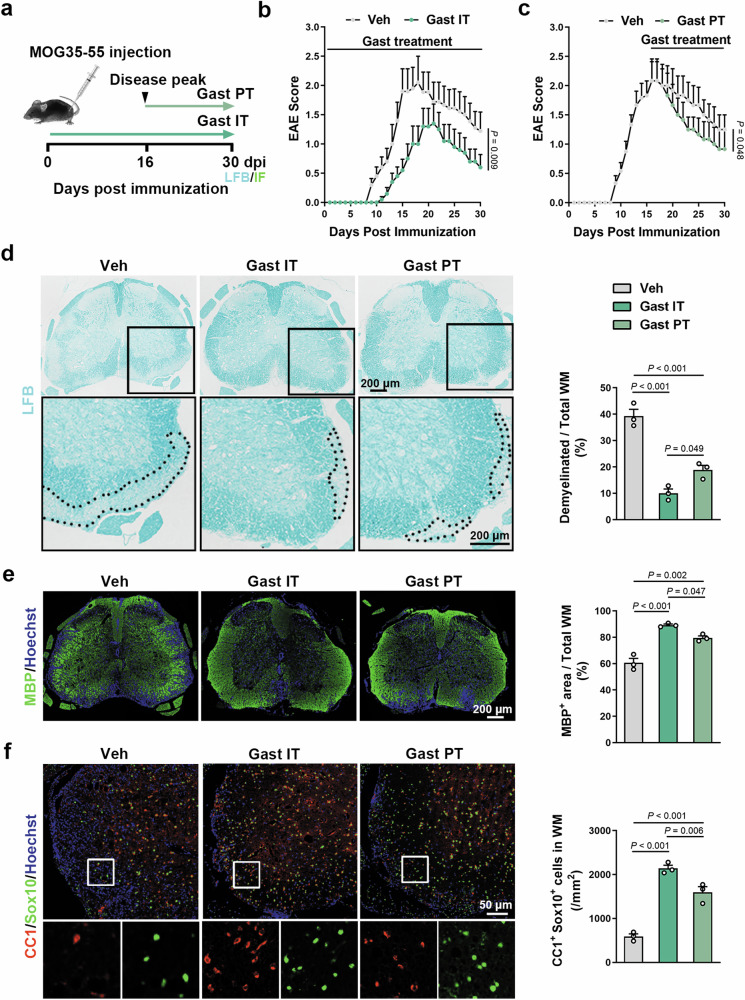
Gastrodin treatment effectively enhances remyelination in EAE mice. **a** Schematic depicting EAE model and gastrodin treatment (gastrodin setting, 100 mg/kg ip). IT: initial treatment, PT: Peak treatment. **b**, **c** Clinical score in EAE mice treated at 1 dpi (left) or treated at disease peak (right) with gastrodin or vehicle (*n* = 6–10 mice). **d** LFB staining of spinal cord sections at 30 dpi (left) and quantification of the demyelinated white matter’s percentage (right, *n* = 3 mice). Black boxes zoom in are shown below. **e** Representative images of MBP (green) immunofluorescence in spinal cord sections at 30 dpi (left) and quantification of MBP^+^ area’s percentage (right, *n* = 3 mice). **f** Representative images of CC1 (red) and Sox10 (green) immunofluorescence around lesions at 30 dpi (left), and quantification of CC1^+^Sox10^+^ cells/mm^2^ (right, *n* = 3 mice). White boxes zoom in are shown below. Data are represented as mean ± SEM, Mann–Whitney U in **b** and **c**, one-way ANOVA with Tukey in **d**–**f**.

Experimental autoimmune encephalomyelitis (EAE) is a commonly used experimental model for MS [48]. To explore the impact of gastrodin in the EAE model, we treated EAE mice with 100 mg/kg gastrodin daily beginning on either one day post-immunization (dpi) or disease peak with the highest clinical score (Fig. 6a). We recorded the clinical scores of the mice daily and found that initial treatment of gastrodin delayed the onset of symptoms mainly ranging from tail weakness to hindlimb paralysis and effectively suppressed the severity of EAE (Fig. 6b). When gastrodin was administered from the disease peak, the severity of EAE was also lower in gastrodin-treated mice than in controls (Fig. 6c). Notably, smaller demyelinating lesions were observed in the lumbar spinal cord of gastrodin-treated mice (Fig. 6d), consistent with a higher proportion of MBP^+^ area after gastrodin treatment (Fig. 6e). Moreover, the extent of remyelination was higher in gastrodin-treated mice correlating with an increased number of CC1^+^Sox10^+^ mature OLs at 30 dpi (Fig. 6f). Thus, gastrodin treatment alleviates immune-induced demyelination damage and enhances remyelination.

## Discussion

Demyelination is one of the typical pathological manifestations of numerous CNS disorders such as MS. Although endogenous remyelination often occurs following demyelination, the process often fails primarily due to two barriers: (1) impaired differentiation of OPCs and (2) reduced myelin generation capacity [6, 49]. As the pivotal cells for remyelination, OL lineage cells have become a major target for promoting myelin repair [50]. Several medications aimed at enhancing OPC differentiation have been tested in patients with MS, including clemastine (a first-generation antihistamine) [10], opicinumab (a human monoclonal antibody against LINGO-1) [51], and bexarotene (a clinically approved RXR agonist) [52]. Although some of them have shown efficacy, they are not as effective as expected. None of them has been approved clinically [53]. One reason may be that the complex microenvironment at the demyelinating lesion of patients is not conducive to OPC differentiation. Several evidences also indicate large number of residual OLs in MS lesions failing to properly remyelinate axons [54, 55]. A recent study demonstrated that therapeutic reversal of epigenetic silencing to reactivate myelin production by mature or newly formed OLs may be necessary to enable myelin regeneration [6]. Therefore, it is essential to develop screening strategies based on enhancing myelinogenic capacity.

Zebrafish, as a vertebrate, well retains cellular functions comparable to those of mammals [56]. Using zebrafish as a screening model in this study was took of their transparency during juvenile stages and their skin permeability which enables direct absorption of small molecule compounds [57, 58]. Furthermore, several different transgenic lines were used to allow dynamic observation of OPC migration, maturation and myelination. In particular, the microinjection of a fluorescent plasmid targeting OLs allowed the observation of myelin development in single OL. Unlike previous cellular level screening compounds, we used the Tg(MBP:eGFP-CAAX) larval as well as the Sox10:mRFP plasmid to ultimately assess the degree of myelination in vivo rather than the degree of OPC differentiation. Using this model, we successfully screened gastrodin as a pro-myelinating candidate from five TCM monomers.

Gastrodin, a compound purified from the notable herbal plant *Gastrodia elata* Blume, is able to pass through the blood-brain barrier and is rapidly distributed in the brain after it enters the systematic circulation [59]. Nowadays, gastrodin has been clinically used as an adjuvant therapy for several neurological disorders such as refractory epilepsy, Parkinson’s, and post-stroke depression [60]. The various effects of gastrodin including mitigating oxidative stress, anti-neuroinflammation, neurotrophic effects and inhibition of neuronal apoptosis would underlie its neuroprotective functions [61–63]. Previous study has reported that gastrodin can increase CNS remyelination by increasing OPC differentiation [64]. It has also been reported that gastrodin significantly enhanced the proliferation, migration and myelination of Schwann cells [65]. In comparison, based on dynamic observations in zebrafish, our findings indicated that gastrodin predominantly stimulated the production of myelin fragments from individual OL without affecting the number of mature OLs. Similarly, although in vitro experiments demonstrated that gastrodin enhanced the proportion of CC1^+^ cells during differentiation, there was no longer a discernible difference in the number of MBP^+^ cells at the subsequent stages of differentiation. Instead, gastrodin mainly affected the branching complexity of mature OLs.

Herein, we demonstrated that gastrodin enhanced myelination via activation of the PI3K/AKT/mTOR signaling pathway in OLs. Activation of PI3K phosphorylates phosphatidylinositol 4,5-bisphosphate (PIP2) to form phosphatidylinositol 3,4,5-trisphosphate (PIP3), which then phosphorylates the serine-threonine kinase AKT, activating mTORC1 [66]. mTORC1 regulates ribosomal protein S6 kinase beta-1 and sterol regulatory element-binding proteins, which are essential transcription factors for the expression of myelin proteins and enzymes involved in lipid synthesis [67]. Previous studies have reported that gastrodin exerts neuroprotective effects through activating the PI3K/AKT signaling pathway in microglia, astrocytes, Schwann cells, and retinal precursor cells [68–71]. In this study, we found that gastrodin significantly increased the phosphorylation level of PI3K and its downstream proteins in OLs, consequently enhancing the expression of myelin proteins. This effect could be effectively impeded by the PI3K inhibitor LY294002. It is worth noting that through computational predictions and biological evidence, gastrodin had a strong binding effect with PI3K, implying that the activation of PI3K/AKT signaling pathway by gastrodin may be related to its direct binding to PI3K. Further investigation is required to ascertain whether this binding is necessary to increase the level of PI3K phosphorylation.

In recent years, several emerging therapeutic strategies have been developed to promote myelination. For instance, the modulation of neuron-oligodendrocyte interactions induces myelination, known as adaptive myelination. Erin M. Gibson et al. have found that optogenetic activation of the premotor cortex can promote oligodendrogenesis and improve myelination in the corpus callosum [72]. Based on this finding, repeated optogenetic activation of demyelinated axons in the motor cortex promotes remyelination and functional restoration of conduction [73]. It has also been reported that activating glutamatergic neurons in the medial prefrontal cortex using optogenetics and chemical genetics promotes the differentiation of OPCs in the corpus callosum, which improves myelin repair and working memory [74]. However, these invasive approaches have not yet reached the stage of clinical implementation, unlike pharmacological pro-myelination strategies. In addition, some therapies that target improvement of the pathological microenvironment, particularly neuroinflammation can also promote remyelination. For example, myelin debris impedes OPC differentiation, and niacin (vitamin B3) promotes myelin debris clearance in lesions by peripheral-derived macrophages and microglia [75]. Moreover, microglia and macrophages synthesize desmosterol which can activate liver X receptor to resolve inflammation and create a permissive environment for OPC differentiation, suggesting that pharmacological stimulation of sterol synthesis has the potential to boost the repair of demyelinated lesions [76]. The anti-inflammatory effects of gastrodin have been highlighted in several prior studies [29, 77–79]. Our findings indicated that gastrodin effectively reduced inflammation while ameliorating myelin defects in the LPC and EAE models, with a milder activation of microglia and astrocytes at the lesions after gastrodin treatment (Supplementary Fig. S5a–e). The anti-inflammatory mechanism of gastrodin in demyelinating diseases remains unclear, yet this aspect warrants further investigation. A dual effect of gastrodin, promoting myelinogenesis and anti-inflammation, strongly indicates the therapeutical potential for the treatment of MS, especially at the chronic-progressive stage.

**Fig. 7:**
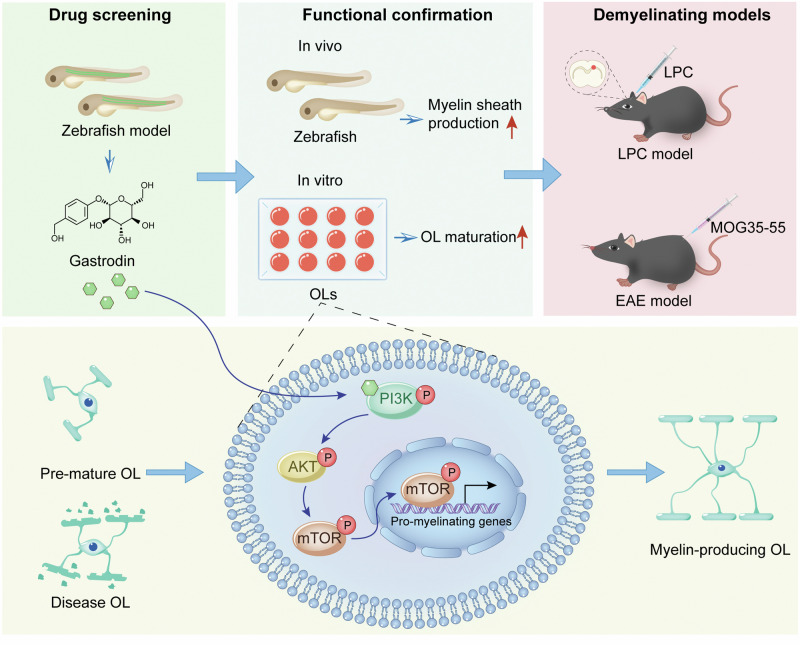
Schematic illustration of drug screening and pro-myelinogenesis effect of gastrodin via PI3K/AKT/mTOR signaling pathway.

In summary, our study provides a great promise in zebrafish screening model for pro-myelination drugs and suggests gastrodin as a potential agent for the treatment of demyelinating diseases (Fig. 7).

## Supplementary information

Supplementary Figure S1

Supplementary Figure S2

Supplementary Figure S3

Supplementary Figure S4

Supplementary Figure S5

Supplementary Figures legend
