# Supplementary figures and images for: Gastrodin promotes CNS myelinogenesis and alleviates demyelinating injury by activating the PI3K/AKT/mTOR signaling
Source: Acta Pharmacol Sin. 2025 Feb 26;46(6):1610–23. doi: 10.1038/s41401-025-01492-z (PMC12098701; doi:10.1038/s41401-025-01492-z)

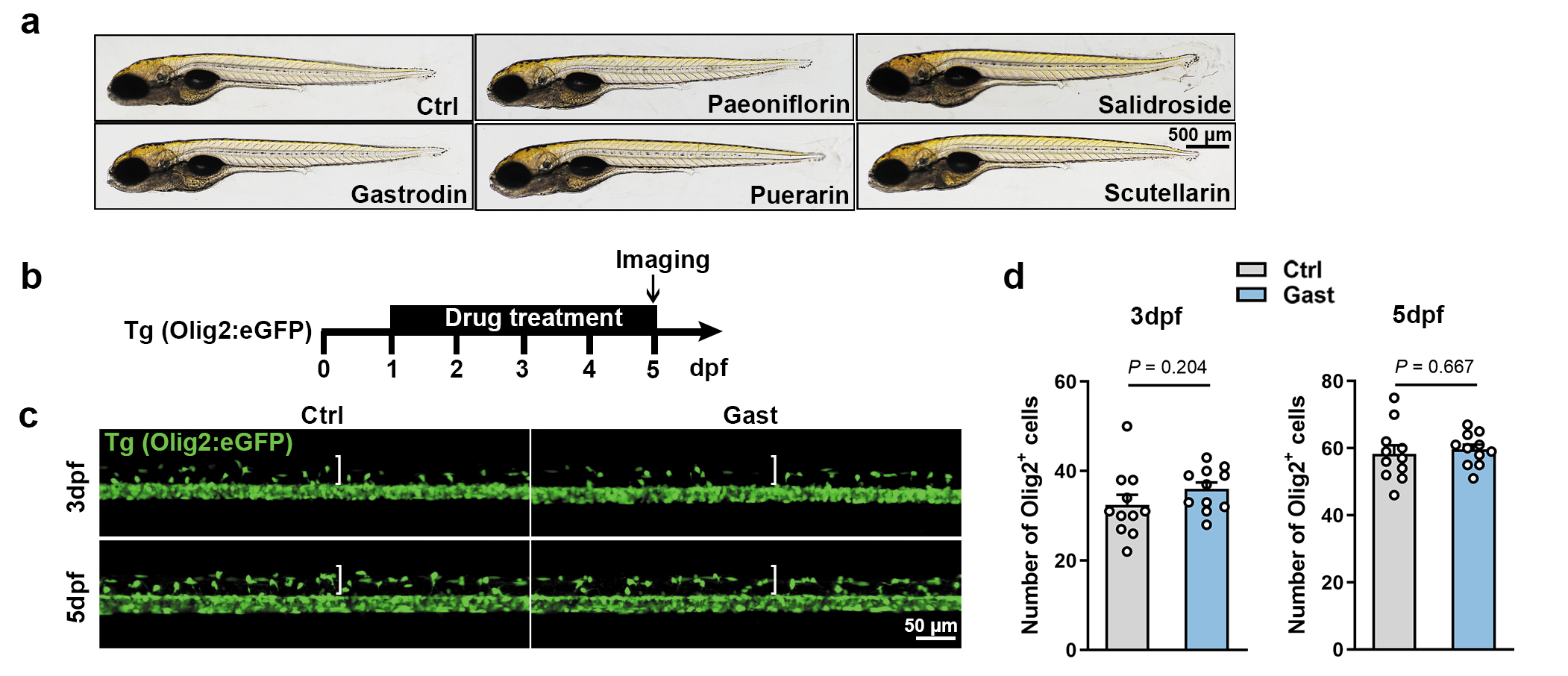

Supplement: Supplementary file 1 — Supplementary Figure S1 [file 41401_2025_1492_MOESM1_ESM.jpg]

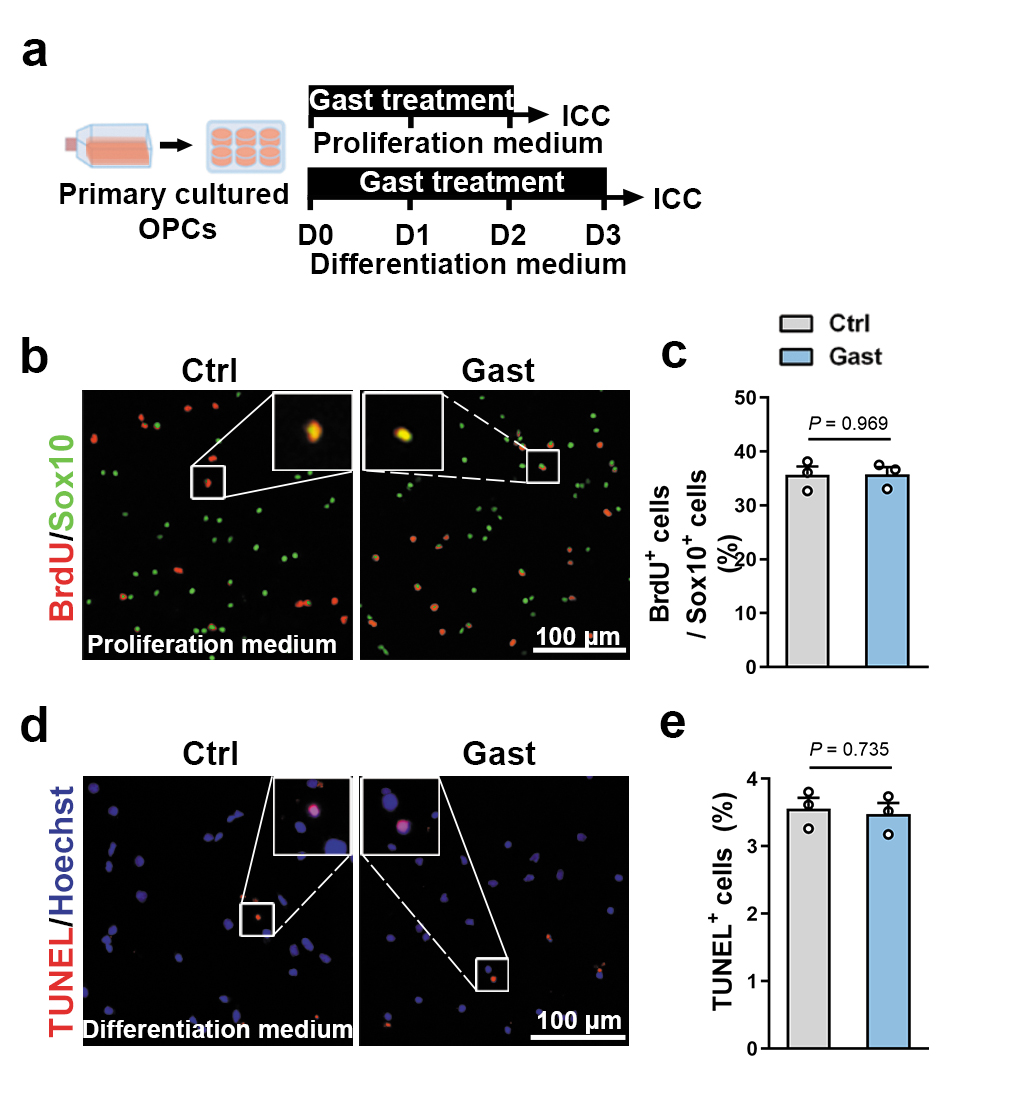

Supplement: Supplementary file 2 — Supplementary Figure S2 [file 41401_2025_1492_MOESM2_ESM.jpg]

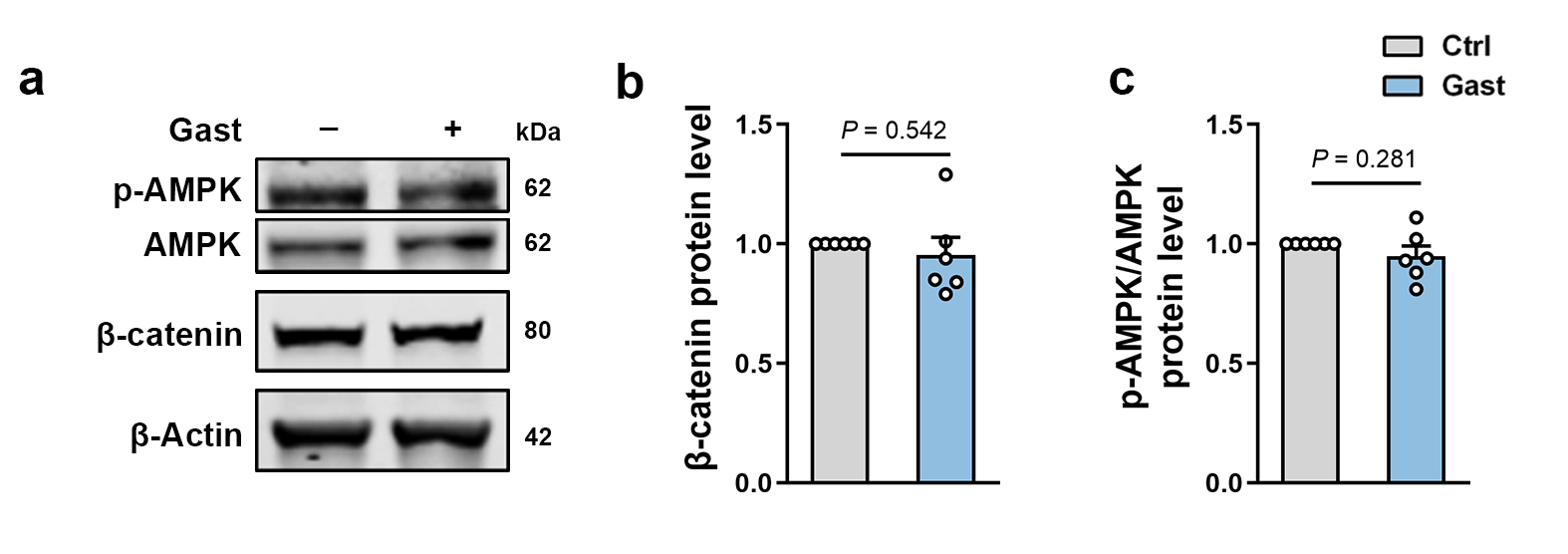

Supplement: Supplementary file 3 — Supplementary Figure S3 [file 41401_2025_1492_MOESM3_ESM.jpg]

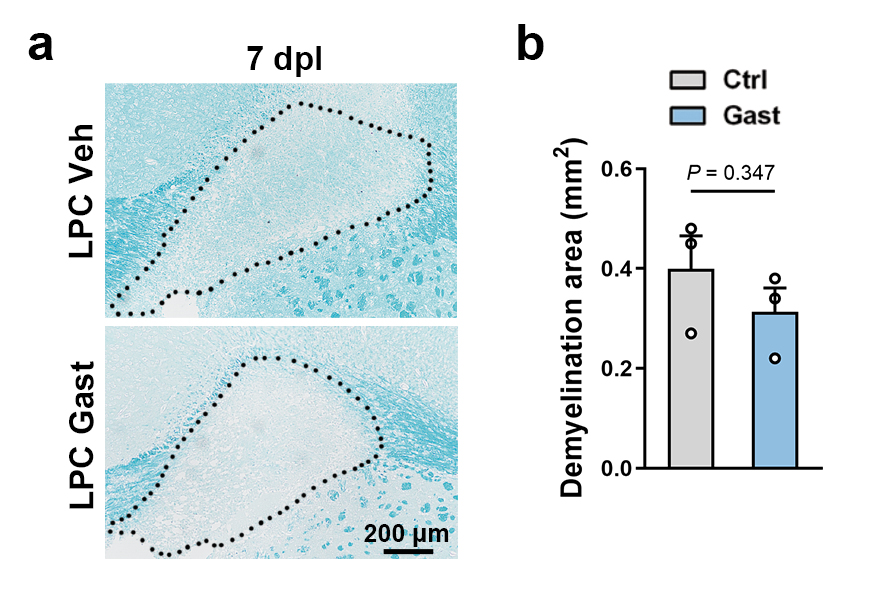

Supplement: Supplementary file 4 — Supplementary Figure S4 [file 41401_2025_1492_MOESM4_ESM.jpg]

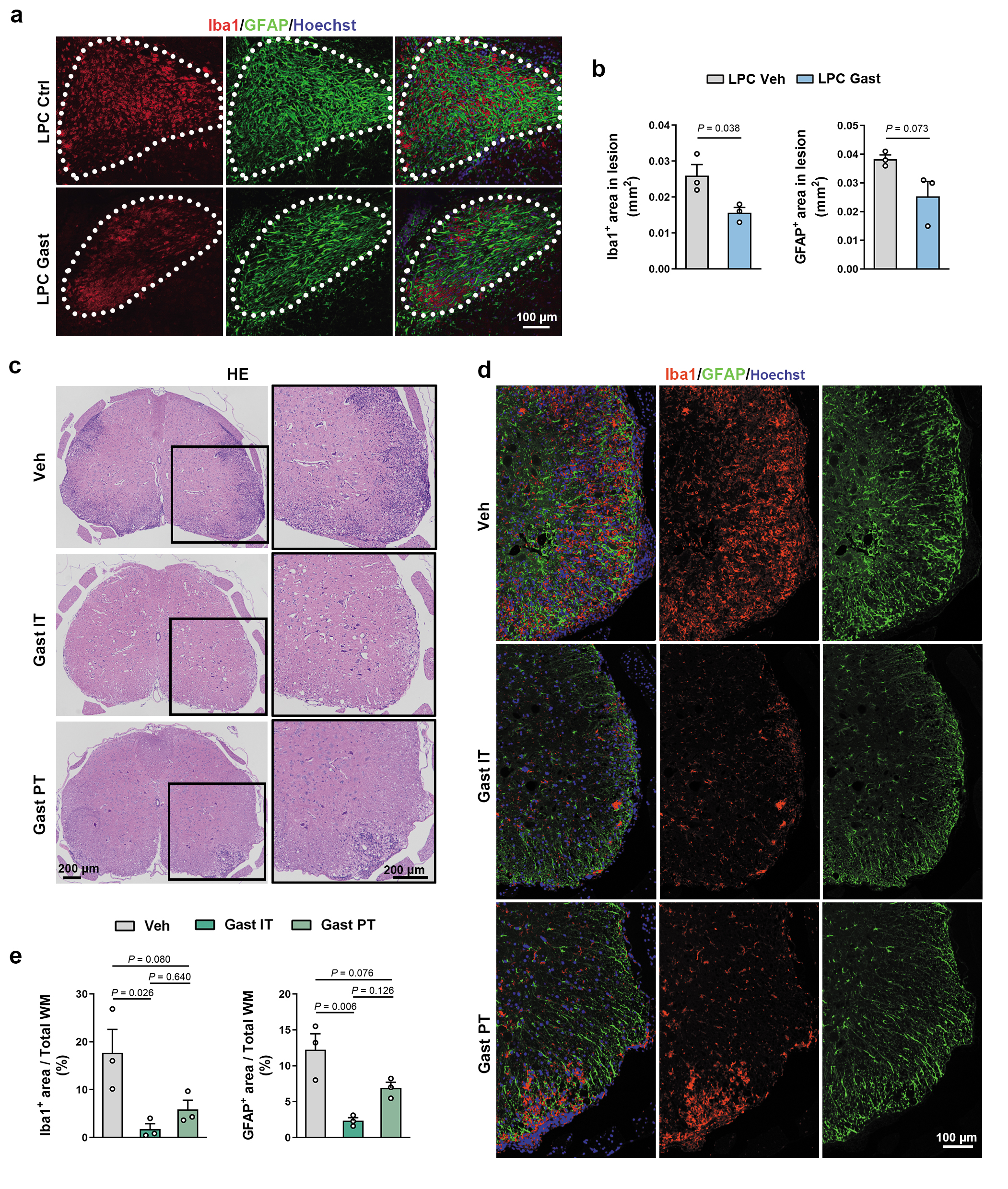

Supplement: Supplementary file 5 — Supplementary Figure S5 [file 41401_2025_1492_MOESM5_ESM.jpg]
