# Supplementary material for: Gastrodin promotes CNS myelinogenesis and alleviates demyelinating injury by activating the PI3K/AKT/mTOR signaling
Source: Acta Pharmacol Sin. 2025 Feb 26;46(6):1610–23. doi: 10.1038/s41401-025-01492-z (PMC12098701; doi:10.1038/s41401-025-01492-z)
Supplement: Supplementary file 6 — Supplementary Figures legend [file 41401_2025_1492_MOESM6_ESM.docx]

**Fig. S1. Gastrodin treatment does not affect the number of OL lineage cells.**

(a) Brightfield images of embryonic zebrafish with control and TCM monomers-treated at 5 dpf. (b) Schematic of Tg(Olig2:eGFP) line imaging pipeline. (c) Representative images showing dorsal Olig2^+^ cells in the Tg(Olig2:eGFP) line at 3 dpf and 5 dpf. (d) Quantification of Olig2^+^ cells number in the dorsal spinal cord (as the white brackets shown) at 3 dpf and 5 dpf (*n* = 11 zebrafish). Data are represented as mean ± SEM, unpaired Student’s *t*-test in (d).

**Fig. S2. Gastrodin does not affect OPC proliferation or apoptosis.**

(a) Schematic depicting OPCs culture and gastrodin treatment. (b) Representative images of BrdU (red) and Sox10 (green) immunofluorescence, amplifying image shows BrdU^+^Sox10^+^ proliferating cells. (c) Quantification of proportion of BrdU^+^Sox10^+^ cells (*n* = 3 experiments). (d) Representative images of TUNEL (red) immunofluorescence, amplifying image shows TUNEL^+^ apoptotic cells. (e) Quantification of TUNEL^+^ cells (*n* = 3 experiments). Data are represented as mean ± SEM, unpaired Student’s *t*-test in (c and e).

**Fig. S3. Gastrodin does not affect the levels of β-catenin and AMPK phosphorylation.**

(a) Western blot for p-AMPK, AMPK, β-catenin. (b) Quantification of the β-catenin

protein level. (c) Quantification of the ratio of p-AMPK/AMPK (*n* = 6 experiments). Data are represented as mean ± SEM, unpaired Student’s *t*-test in (b and c).

**Fig. S4. Gastrodin does not affect LPC-induced early demyelinating lesions.**

(a) LFB staining showing the demyelinated region in the corpus callosum at 7 dpl. (b) Quantification of the demyelinated area at 7 dpl (*n* = 3 mice). Data are represented as mean ± SEM, unpaired Student’s *t*-test in (b).

**Fig. S5. Gastrodin alleviate the inflammation of LPC and EAE models.**

(a) Representative images of Iba1 (red) and GFAP (green) immunofluorescence in LPC-lesioned corpus callosum at 14 dpl. Inflammation-activated areas are shown in the white dotted line. (b) Quantification of Iba1^+^ and GFAP^+^ areas in the LPC lesion (*n* = 3 mice). (c) Representative images of H&E staining in EAE spinal cord sections. Gastrodin treatment reduced immune cell infiltration. (d) Representative images of Iba1 (red) and GFAP (green) immunofluorescence in lesions of EAE mice at 30 dpi. (e) Quantification of Iba1^+^ and GFAP^+^ areas in lesions of EAE mice (*n* = 3 mice). Data are represented as mean ± SEM, unpaired Student’s *t*-test in (b), one-way ANOVA with Tukey in (e).
